# Supplementary material for: Metataxonomic Identification of Microorganisms during the Coffee Fermentation Process in Colombian Farms (Cesar Department)
Source: Foods. 2024 Mar 9;13(6):839. doi: 10.3390/foods13060839 (PMC10969282; doi:10.3390/foods13060839)
Supplement: Supplementary file 1 [file foods-13-00839-s001.zip › foods-2844642-supplementary.pdf]

Supplementary Material

Metataxonomic and Microbial Characterization of the Coffee Fermentation Process in Colombian Farms (Cesar Department)

Carmenza E. Góngora[1*], Laura Holguín-Sterling[2], Bertilda Pedraza-Claros[2-3], Rosangela Perez-Salinas[3], Aristóteles Ortiz[4], Lucio Navarro-Escalante[1-5].

1 Department of Entomology. 2 Department of Post Harvesting. 4 Department of Plant Physiology. National Coffee Research Center, Cenicafé. Manizales 170009, Colombia;
carmenza.gongora@cafedecolombia.com (C.E.G.); lhsterling22@gmail.com (L.H.S.); aristofeles.ortiz@cafedecolombia.com (A.O.)

3 Cienciaudes Research Group, Faculty of Health Science, Universidad de Santander. Valledupar 200004, Colombia; ber.pedraza@mail.udes.edu.co (B.P.C.); ros.perez@mail.udes.edu.co (R.P.S.)

5 Current position: Department of Molecular Biosciences, University of Texas at Austin, Austin TX 78712; lucio.navarro@austin.utexas.edu; lucio.navarro@austin.utexas.edu (L.N.-E.)

Supplementary Table S1. Technical characteristics of the farms from the department of Cesar.

Farm	Municipality	Georegistration		Coffee variety	Fermentation characteristics	
		Coordinates	Altitude (m.a.s.l)		Time (h)	Condition
F1	Pueblo Bello	10°24.2225'N-73°,662268'O	1390	Castillo General	36	Fermentation without water, tiled tank
F2	Pueblo Bello	10°21.6800'N-73°43.2610'O	1700	Castillo General	36	Fermentation without water, tiled tank
F3	La Paz	10°19.1600'N -72°59.6350'O	1820	Castillo General	20	Fermentation without water, plastic tank
F4	La jagua de Ibirico	9°31,1220 N-73° 10.9080'O	1835	Castillo General	18	Fermentation with water, tiled tank
F5	Agustín Codazzi	9°32.4960'N-73° 9.6120'O	1492	Colombia	18	Fermentation with water, concrete tank
F6	Agustín Codazzi	9°58.6670'N-73°2.8470'O	1813	Colombia	42	Fermentation without water, tiled tank
F7	Pueblo Bello	10°22.2000'N-73°42.1280'O	1588	Cenicafé 1	36	Submerged fermentation in plastic tanks with lids
F8	Pueblo Bello	10°22.19'N-73°42'16'O	1540	Colombia	18	Fermentation without water, tiled tank
F9	Agustín Codazzi	10°2.2530'N-73°7.9449'O	1261	Castillo General	18	Fermentation without water, plastic tank
F10	Agustín Codazzi	10°2.2550'N-73°8.3970'O	1124	Colombia	18	Fermentation with water, concrete tank
F11	La Paz	10°15.9740' N-73°4.7040'O	1116	Colombia	36	Fermentation with water, concrete tank
F12	La Paz	10°16.2290'N-73°2.8720'O	1518	Castillo General	14	Fermentation with water, concrete tank
F13	Pueblo Bello	10°22.59'N-73°3870'O	1162	Colombia	16	Fermentation without water, concrete tank
F14	Pueblo Bello	10°235.38'N-73°37'30.761'O	1123	Castillo General	10	Fermentation without water, concrete tank
F15	La Paz	10°20.6400'N-72°57.2930'O	1837	Colombia	16	Fermentation without water, tiled tank
F16	La Paz	10°20.7120'N-72°57.2620'O	1938	Castillo General	16	Fermentation with water, tiled tank
F17	La Paz	10°20.3640'N-72°57.3940'O	1616	Castillo General	16	Fermentation with water, concrete and tiled tank
F18	Pueblo Bello	10°22.5830'N-73°34.3244'O	1407	Colombia	18	Fermentation with water, tiled tank
F19	La jagua de Ibirico	09°32.199'N-73°10.319'O	1623	Castillo General	18	Fermentation with water, tiled tank
F20	La jagua de Ibirico	09°31.784'N-73°10.380'O	1740	Castillo General	18	Fermentation without water, concrete tank

Supplementary Table S2. Coffee Harvest Characterization from 20 Farms whithin the Department of Cesar, Colombia.

Farm	Sample	1 (Green)*	2 (bright green)*	3 (bright)*	4 (Ripe 1)*	5 (Ripe 2)*	6 (Overripe 1)*	7 (Overripe 2)*	8 (Dry)*	Total	Percentage	Category
F1	Fruits	1	11	14	22	30	46	74	16	214	0.38%	Excelent
	weight (g)	1	13	22	33	50	77	132	11	339		
F2	Fruits	4	5	16	17	58	56	22	3	181	1.51%	Excelent
	weight (g)	3.1	6	30	32	108	110	34	2	325.1		
F3	Fruits	2	15	19	22	70	49	20	6	203	0.75%	Excelent
	weight (g)	3	21.6	31.4	39.6	127.4	100.1	41	4.5	368.6		
F4	Fruits	5	9	20	20	60	55	28	9	206	1.88%	Excelent
	weight (g)	5.4	9.6	31.6	31.6	92.5	100.9	46.9	8.7	327.2		
F5	Fruits	8	4	4	18	26	65	98	7	230	3.01%	Good
	weight (g)	9.7	5.9	7.4	31	46	113.7	158.5	5.1	377.3		
F6	Fruits	4	-	13	15	48	23	90	10	203	1.51%	Excelent
	weight (g)	6.7	-	18.4	24	85.5	40.9	150.6	9.8	335.9		
F7	Fruits	1	10	16	61	55	40	21	18	222	0.38%	Excelent
	weight (g)	1.3	13.7	25.3	93.1	84.4	76.8	44.4	19.8	358.8		
F8	Fruits	-	7	30	38	36	67	24	25	227	0	Excelent
	weight (g)	-	7.1	28.5	42.1	45.8	79.8	29.8	19.4	252.5		
F9	Fruits	4	26	33	48	54	31	30	20	246	1.51%	Excelent
	weight (g)	7.6	41.3	53.1	76.8	87.1	56.5	48.5	18.2	389.1		
F10	Fruits	8	22	31	44	59	31	29	14	238	3.01%	Good
	weight (g)	13.9	34.7	48.3	69.4	90.8	49.8	48.1	12.8	367.8		
F11	Fruits	3	10	13	24	42	38	23	15	168	1.13%	Excelent
	weight (g)	3.2	13.4	29.8	39.4	69.1	66.3	38.6	12.8	272.6		
F12	Fruits	6	8	9	19	35	38	36	23	174	2.26%	Good
	weight (g)	9.1	15.4	16.8	32.7	58.5	63.1	59.6	17.4	272.6		
F13	Fruits	4	6	13	21	33	38	36	26	177	1.51%	Excelent
	weight (g)	4.7	11.8	23.4	35.8	57.1	63.4	61.7	20.4	278.3		
F14	Fruits	3	7	18	22	36	35	38	9	168	1.13%	Excelent
	weight (g)	3.2	13.5	33.4	37.1	59.8	60.1	63.7	7.4	278.2		
F15	Fruits	5	38	25	36	66	35	5	12	222	1.88%	Excelent
	weight (g)	4.72	47	41	61	116	67	9.56	14.34	360.6		
F16	Fruits	7	16	53	48	33	44	14	8	223	2.63%	Good
	weight (g)	12.15	22	77	79	55	82	28	7	362.2		
F17	Fruits	4	1	14	16	66	35	43	44	223	1.51%	Excelent
	weight (g)	3.37	1.27	17	24	108	74	75	44	346.6		
F18	Fruits	19	9	10	-	144	-	14	30	226	7.53%	Medium
	weight (g)	22.87	12.41	16.85	-	230	-	21	20.13	323.3		
F19	Fruits	-	7	27	43	125	12	15	6	235	0%	Excelent
	weight (g)	-	10	43	84	202	18	6	5	368		
F20	Fruits	3	44	22	39	37	15	6	63	229	1.13%	Excelent
	weight (g)	2.63	57	36	64	78	29	10	32	308.6		

*According color chart Cromacafé (Peñuela-Martínez. Guerrero and Sanz-Uribe. 2022)

Peñuela-Martínez. A. E.. Guerrero. Á.. & Sanz-Uribe. J. R. (2022). Cromacafé® Herramienta para identificar los estados de madurez de las variedades de café de fruto rojo. *Avances Técnicos Cenicafé*. *535*. 1-8. https://doi.org/10.38141/10779/0535

Supplementary Table S3. Physico-chemical results of pH, Brix degrees, and total acidity of the mucilage throughout the coffee fermentation process in 20 farms from the Department of Cesar, Colombia.

Farm	Time (h)	pH	Brix degrees (°Bx)	Total acidiy (mg L^-1 CaCo3)	Sucrose (%)	Reducing sugars (%)
F1	0	4.34 ± 0.54	5.00 ± 0.00	248.4 ± 30.00	0.04 ± 0.01	3.32 ± 0.77
	18	3.30 ± 0.26	5.00 ± 0.00	538.4 ± 30.00	0.05 ± 0.02	1.78 ± 0.35
	36	3.30 ± 0.26	4.00 ± 0.00	741.7 ± 75.10	ND	0.38 ± 0.15
F2	0	3.80 ± 0.27	4.50 ± 0.50	333.4 ± 65.00	0.03 ± 0.01	1.66 ± 0.81
	18	3.90 ± 0.09	3.00 ± 0.00	408.4 ± 26.50	ND	0.04 ± 0.01
	36	3.97 ± 0.06	3.00 ± 0.00	408.4 ± 10.00	0.03 ± 0.01	0.04 ± 0.02
F3	0	5.34 ± 0.30	11.67 ± 0.29	153.4 ± 13.20	0.20 ± 0.04	1.02 ± 0.21
	10	4.33 ± 0.30	7.83 ± 0.29	265.1 ± 15.30	0.06 ± 0.02	0.61 ± 0.32
	20	4.06 ± 0.06	4.00 ± 0.00	203.4 ± 8.70	0.15 ± 0.06	3.24 ± 0.02
F4	0	5.39 ± 0.36	10.00 ± 0.00	128.4 ± 10.00	0.05 ± 0.01	7.24 ± 1.58
	9	5.34 ± 0.29	8.17 ± 0.29	211.7 ± 15.30	0.03 ± 0.01	4.09 ± 0.09
	18	5.39 ± 0.21	6.00 ± 0.00	208.4 ± 10.00	0.05 ± 0.01	3.92 ± 0.77
F5	0	5.16 ± 0.17	3.67 ± 0.58	68.4 ± 10.00	0.75 ± 0.01	3.50 ± 1.08
	9	4.84 ± 0.14	4.67 ± 0.76	123.4 ± 13.20	0.29 ± 0.09	1.33 ± 0.09
	18	4.29 ± 0.26	4.00 ± 0.00	271.7 ± 5.80	ND	1.51 ± 0.03
F6	0	4.84 ± 0.20	6.00 ± 1.00	73.4 ± 5.00	0.45 ± 0.04	4.09 ± 1.08
	21	3.17 ± 0.15	6.17 ± 0.29	351.7 ± 32.10	0.24 ± 0.06	1.93 ± 0.70
	42	3.20 ± 0.17	4.00 ± 0.00	331.7 ± 15.30	0.05 ± 0.01	0.53 ± 0.13
F7	0	4.52 ± 0.11	2.70 ± 0.00	197.5 ± 2.90	ND	0.48 ± 0.20
	18	4.20 ± 1.40	2.93 ± 0.06	289.7 ± 0.60	0.03 ± 0.01	0.08 ± 0.03
	36	4.03 ± 0.01	2.63 ± 0.12	307.1 ± 3.20	0.03 ± 0.01	0.03 ± 0.00
F8	0	5.31 ± 0.03	6.03 ± 0.06	41.6 ± 0.50	0.75 ± 0.09	4.26 ± 1.39
	9	4.24 ± 0.02	4.30 ± 0.10	101.1 ± 3.60	0.05 ± 0.06	1.49 ± 0.59
	18	3.52 ± 0.02	2.87 ± 0.06	217.0 ± 4.00	0.03 ± 0.05	0.31 ± 0.15
F9	0	4.76 ± 0.02	4.80 ± 0.00	22.2 ± 1.20	0.28 ± 0.06	2.46 ± 0.14
	9	3.95 ± 0.01	4.20 ± 0.17	95.5 ± 1.80	0.18 ± 0.01	2.19 ± 0.77
	18	3.35 ± 0.00	3.93 ± 0.06	224.3 ± 4.60	0.05 ± 0.01	0.66 ± 0.33
F10	0	5.08 ± 0.01	3.23 ± 0.06	11.6 ± 0.90	0.06 ± 0.01	1.28 ± 0.50
	9	4.65 ± 0.01	2.37 ± 0.06	36.0 ± 1.60	0.02 ± 0.01	0.29 ± 0.13
	18	4.04 ± 0.01	1.27 ± 0.06	32.5 ± 0.90	0.02 ± 0.01	0.04 ± 0.02
F11	0	5.08 ± 0.05	4.07 ± 0.06	19.6 ± 0.80	0.11 ± 0.04	2.47 ± 1.11
	18	3.50 ± 0.01	3.03 ± 0.32	194.2 ± 5.30	0.03 ± 0.01	0.26 ± 0.12
	36	4.32 ± 0.03	3.00 ± 0.10	100.5 ± 1.00	0.02 ± 0.01	0.02 ± 0.01
F12	0	5.39 ± 0.02	3.50 ± 0.00	5.3 ± 0.20	0.61 ± 0.06	2.19 ± 0.59
	7	4.87 ± 0.01	2.07 ± 0.06	31.4 ± 1.70	0.06 ± 0.02	1.02 ± 0.34
	14	3.69 ± 0.01	2.00 ± 0.00	84.5 ± 2.60	0.03 ± 0.01	0.34 ± 0.05
F13	0	4.98 ± 0.01	5.13 ± 0.23	103.6 ± 2.90	0.75 ± 0.10	3.27 ± 0.32
	8	4.31 ± 0.01	3.80 ± 0.10	198.3 ± 1.30	0.18 ± 0.08	2.64 ± 0.60
	16	3.48 ± 0.01	3.23 ± 0.15	423.8 ± 4.40	0.13 ± 0.04	1.45 ± 0.29
F14	0	5.19 ± 0.03	8.23 ± 0.23	129.0 ± 7.60	0.49 ± 0.05	5.79 ± 0.89

	5	4.81	±	0.01	5.67	±	0.55	146.9	±	1.80	0.14	±	0.01	4.01	±	0.50
	10	4.19	±	0.02	5.00	±	0.00	282.7	±	21.20	0.03	±	0.01	3.02	±	0.27
F15	0	5.69	±	0.02	3.80	±	0.00	56.9	±	2.60	0.26	±	0.01	2.63	±	0.14
	8	5.05	±	0.03	3.27	±	0.15	74.8	±	4.90	0.09	±	0.04	2.35	±	0.31
	16	3.66	±	0.01	3.03	±	0.06	134.9	±	14.10	0.13	±	0.06	1.74	±	0.20
F16	0	5.75	±	0.02	3.03	±	0.06	25.5	±	1.50	0.11	±	0.04	2.13	±	0.11
	8	5.34	±	0.00	2.77	±	0.15	50.3	±	1.50	0.13	±	0.06	1.92	±	0.32
	16	3.95	±	0.00	2.83	±	0.12	115.6	±	5.00	0.09	±	0.01	1.29	±	0.52
F17	0	5.30	±	0.02	3.37	±	0.15	57.5	±	3.00	0.04	±	0.01	2.21	±	0.04
	8	4.83	±	0.02	4.30	±	0.10	86.7	±	1.60	0.01	±	0.00	2.14	±	0.39
	16	3.78	±	0.01	3.53	±	0.12	165.8	±	5.90	0.01	±	0.00	1.37	±	0.31
F18	0	3.89	±	0.01	3.60	±	0.20	117.9	±	14.00	0.04	±	0.01	1.09	±	0.61
	9	4.26	±	0.02	3.40	±	0.10	49.7	±	7.00	0.11	±	0.40	2.31	±	0.07
	18	3.68	±	0.02	4.20	±	0.10	120.0	±	3.30	0.23	±	0.04	2.17	±	0.42
F19	0	4.84	±	0.03	2.20	±	0.10	4.1	±	2.40		ND		1.38	±	0.13
	9	4.76	±	0.06	2.93	±	0.06	28.5	±	4.30	0.01	±	0.00	1.63	±	0.55
	18	3.28	±	0.02	3.90	±	0.30	120.5	±	8.50	0.02	±	0.01	0.53	±	0.28
F20	0	4.66	±	0.02	3.23	±	0.32	10.2	±	2.60		ND		1.08	±	0.39
	9	4.49	±	0.02	1.70	±	0.26	80.4	±	3.50	0.01	±	0.00	0.30	±	0.10
	18	3.64	±	0.02	2.13	±	0.15	314.7	±	7.70		ND		0.10	±	0.01

*ND: Not Detected

Supplementary Table S4. Microbial counts from the coffee mucilage throughout the coffee fermentation process in 20 farms from the Department of Cesar, Colombia.

Farm	Fermentation Time (h)	Mesophiles	Acid Lactic bacteria (LAB)	Coliforms	Yeast	Mycelial Fungi
		Log 10 cfu/mL				
F1	0	5.23	5.68	3.30	5.40	4.43
	18	5.64	3.30	3.30	5.53	4.38
	36	5.11	3.30	3.00	5.34	4.43
F2	0	5.56	3.30	3.00	5.80	3.30
	18	4.90	3.90	3.00	6.02	3.30
	36	5.61	3.30	3.30	5.74	3.00
F3	0	5.76	6.43	3.00	6.10	3.30
	10	5.76	6.43	3.00	6.00	3.70
	20	5.98	3.30	4.28	5.99	3.00
F4	0	6.05	6.35	5.20	5.99	4.08
	9	5.69	6.24	3.48	5.04	4.00
	18	5.91	6.42	ND	6.02	3.48
F5	0	5.30	6.25	3.70	5.52	3.30
	9	6.00	6.43	4.20	6.15	3.30
	18	6.08	5.94	3.00	5.92	3.00
F6	0	5.74	4.41	4.89	5.90	3.30
	21	6.22	3.60	3.00	6.18	3.70
	42	6.60	3.48	3.00	6.41	3.00
F7	0	5.68	9.51	4.00	5.90	ND
	18	5.75	9.07	3.85	5.86	ND
	36	5.82	8.89	3.78	5.66	ND
F8	0	5.88	8.48	5.45	5.50	ND
	9	5.81	9.09	5.35	5.47	ND
	18	5.79	9.07	5.31	5.65	ND
F9	0	5.62	5.93	5.58	5.87	3.60
	9	6.12	5.97	4.92	5.78	3.60
	18	6.09	5.85	4.63	5.82	ND
F10	0	5.65	5.89	4.93	5.62	3.48
	9	5.53	5.89	5.16	5.48	3.60
	18	5.27	5.87	5.07	5.75	4.08
F11	0	5.65	6.09	4.77	5.74	3.60
	18	5.83	6.02	4.49	5.69	3.30
	36	5.66	5.92	5.22	5.92	3.70
F12	0	5.30	5.99	4.93	5.76	ND
	7	5.69	5.97	3.95	5.72	3.00
	14	5.67	6.01	4.30	5.09	ND
F13	0	5.45	7.14	4.26	5.56	ND
	8	5.21	7.11	ND	5.64	ND

	16	4.59	7.04	ND	5.82	ND
F14	0	5.50	7.18	4.94	5.79	3.70
	5	5.42	7.08	4.41	5.55	3.78
	10	5.65	6.71	4.83	6.02	3.60
F15	0	5.46	5.90	4.00	5.81	3.78
	8	5.25	5.78	4.28	5.77	3.60
	16	5.27	5.90	4.59	5.95	3.30
F16	0	5.59	6.04	4.46	5.90	3.30
	8	5.47	6.03	4.18	5.88	2.70
	16	5.42	5.83	4.60	5.87	3.60
F17	0	5.59	7.14	5.15	5.68	3.60
	8	5.57	7.13	4.54	5.52	3.60
	16	5.52	7.14	4.48	5.65	3.30
F18	0	5.13	7.15	3.85	5.61	3.00
	9	5.02	7.21	4.71	5.42	3.60
	18	5.50	7.23	4.98	5.41	3.60
F19	0	5.48	7.22	4.30	5.52	3.30
	9	4.98	7.29	3.70	5.36	3.30
	18	4.94	7.29	3.00	5.46	3.60
F20	0	5.41	7.12	3.60	5.26	3.70
	9	5.28	7.22	3.30	5.26	3.48
	18	5.29	7.24	ND	5.08	3.00

ND: Non detected

Supplementary Table S5. High throughput sequencing of 16S rRNA gene with the MiSeq platform from Ilumina and richness and diversity indices during coffee fermentation in 20 farms department of Cesar, Colombia.

Farm	Fermentation Time (h)	Read Count	Q30 (%)	nseqs	Coverage	Genus-Species Observed (OTUs)	ACE	Shannon	Simpson
F1	0	189.24	85.50	14098	0.99	337	585.30	2.27	0.79
	18	171.07	86.80	14127	0.99	298	519.70	2.46	0.83
	36	186.24	84.80	14128	0.99	198	344.93	1.97	0.70
F2	0	163.35	85.00	14028	0.99	361	652.07	1.90	0.52
	18	162.28	86.90	14104	0.99	218	398.49	1.91	0.68
	36	192.81	86.90	14112	0.99	244	563.27	1.77	0.65
F3	0	191.89	85.40	14260	0.98	422	1085.03	2.55	0.85
	10	203.32	86.10	14091	0.99	244	397.34	1.92	0.70
	20	174.42	86.60	14102	0.99	189	345.77	1.58	0.63
F4	0	186.57	85.20	14161	0.99	342	507.73	1.77	0.53
	9	195.14	86.80	14112	0.99	183	284.92	2.13	0.79
	18	193.86	85.80	14067	0.99	259	497.14	2.22	0.79

F5	0	202.91	84.30	14198	0.98	488	1144.80	2.41	0.77
	9	175.52	85.60	14237	0.98	502	1141.33	3.01	0.87
	18	206.94	85.80	14116	0.99	219	567.24	2.20	0.81
F6	0	191.56	85.50	14273	0.98	412	1163.83	2.70	0.88
	21	200.77	87.00	14135	0.99	184	377.80	2.10	0.78
	42	153.73	86.40	14115	0.99	172	279.66	2.18	0.79
F7	0	229.43	79.60	18622	0.99	395	674.70	2.50	0.85
	18	247.78	80.90	18721	0.99	305	674.40	2.10	0.80
	36	222.81	80.40	18688	0.99	287	506.20	1.90	0.75
F8	0	227.16	79.00	18953	0.96	1008	256.80	3.70	0.92
	9	233.25	79.60	18576	0.98	598	110.20	3.10	0.90
	18	225.90	79.40	18663	0.98	425	715.80	2.50	0.86
F9	0	237.51	79.30	18915	0.97	940	199.90	4.00	0.95
	9	203.63	78.60	18535	0.98	487	805.00	2.80	0.85
	18	158.63	80.00	18603	0.99	298	544.00	2.30	0.83
F10	0	212.41	79.50	18894	0.97	951	220.10	4.10	0.95
	9	217.30	79.10	18595	0.98	433	762.70	2.40	0.79
	18	197.48	79.90	18601	0.98	436	764.60	2.60	0.82
F11	0	196.53	78.60	18897	0.97	817	252.10	3.70	0.94
	18	211.95	80.10	18676	0.98	377	839.60	2.10	0.78
	36	195.33	80.50	18700	0.99	337	563.80	2.20	0.74
F12	0	202.45	79.50	18913	0.97	765	222.50	2.80	0.84
	7	178.44	79.90	18908	0.97	668	221.00	2.60	0.81
	14	230.04	80.20	18653	0.99	284	586.10	2.20	0.79
F13	0	196.49	79.80	18882	0.97	812	1993.60	3.20	0.87
	8	215.42	81.20	18675	0.98	350	771.20	1.90	0.61
	16	232.86	80.50	18680	0.99	305	695.20	2.40	0.86
F14	0	192.59	79.00	18863	0.96	1002	2762.70	3.80	0.93
	5	167.40	79.10	18906	0.96	906	2701.70	3.50	0.91
	10	176.09	80.30	18556	0.99	366	661.80	2.50	0.84
F15	0	209.90	78.90	18765	0.98	524	869.90	2.90	0.83
	8	237.55	78.70	18965	0.97	843	2180.80	3.00	0.89
	16	230.69	79.30	18675	0.99	269	474.90	2.50	0.86
F16	0	219.79	78.40	18817	0.98	560	853.80	3.20	0.86
	7	188.35	78.80	18788	0.98	546	1449.20	2.80	0.86
	14	201.87	79.90	18650	0.99	273	502.40	2.60	0.88
F17	0	217.16	79.20	18949	0.96	927	2499.40	3.50	0.91
	8	160.10	78.80	18840	0.97	822	2171.70	3.40	0.91
	16	196.06	79.90	18552	0.98	457	807.30	2.70	0.87
F18	0	167.43	79.00	18894	0.97	701	2012.00	3.00	0.90

	9	217.42	79.20	18523	0.98	424	768.80	2.70	0.86
	18	198.37	80.10	18582	0.99	343	615.60	2.70	0.90
F19	0	216.47	79.20	18926	0.97	883	2446.30	3.40	0.91
	9	253.56	80.40	18640	0.98	415	1005.40	2.50	0.82
	18	202.02	79.70	18670	0.99	305	592.10	2.30	0.84
F20	0	119.23	79.10	18857	0.98	767	1187.20	3.80	0.93
	9	178.11	80.20	18937	0.97	877	2150.60	3.30	0.87
	18	209.35	79.50	18669	0.98	431	806.00	2.10	0.79

Supplemetary Table S6. Identification and frequency of mesophilic aerobic bacteria isolated into pure culture in coffee mucilage during the fermentation process at three different times in the 20 studied farms.

Microorganism	Time	Farms																				Frequency by farms	
		F1	F2	F3	F4	F5	F6	F7	F8	F9	F10	F11	F12	F13	F14	F15	F16	F17	F18	F19	F20	#	%
Acinetobacter iwoffii	T3													■								1	5.0
Acinetobacter haemolyticus	T1														■								
	T2																					1	5.0
	T3																						
Acinetobacter calcoacético	T1										■												
	T2																					1	5.0
	T3																						
Acinetobacter baumani	T1								■														
	T2									■										■		3	15.0
	T3																						
Averyella spp.	T1																						
	T2																					1	5.0
	T3				■																		
Averyella dalhousiensis	T1					■																	
	T2						■															2	10.0
	T3						■																
Bacillus spp.	T1																						
	T2		■											■							■	3	15.0
	T3																						
Bacillus subtilis	T1													■									
	T2		■				■															4	20.0
	T3						■							■						■			
Bacillus firmus	T1						■																
	T2	■														■		■		■		5	25.0
	T3																						
Bacillus megaterium	T1																						
	T2																					1	5.0
	T3							■															
Bacillus cereus	T1																						
	T2				■																	1	5.0
	T3																						
Bordetella spp.	T1																						
	T2											■										1	5.0
	T3																						
Citrobacter freudii	T1	■						■															
	T2	■								■												3	15.0
	T3	■																					
Chryseobacterium indologenes	T1					■																	
	T2																					1	5.0
	T3																						
Chromobacterium violaceum	T1																						
	T2																					1	5.0
	T3																			■			
Enterobacter cloacae	T1								■														
	T2					■					■											3	15.0
	T3																						
Enterobacter gergoviae	T1							■															
	T2																					1	5.0
	T3																						
Enterobacter aerogenes	T1											■											
	T2																					2	10.0
	T3							■				■											
	T1												■									2	10.0

Enterobacter taylorae	T2																						
	T3																						
Escherichia coli	T1						■															3	15.0
	T2					■			■														
	T3								■														
Hafnia alvei	T1																■					1	5.0
	T2																						
	T3																						
Klebsiella ornithinolytica	T1																					1	5.0
	T2	■																					
	T3																						
Klebsiella ozaenae	T1				■							■										2	10.0
	T2				■																		
	T3																						
Klebsiella planticola	T1																					1	5.0
	T2																						
	T3			■																			
Klebsiella oxytoca	T1									■												2	10.0
	T2								■														
	T3																						
Klebsiella pneumoniae	T1								■					■								4	20.0
	T2											■	■										
	T3																						
Kluyvera ascorbata	T1			■																		1	5.0
	T2																						
	T3																						
Micrococcus spp.	T1	■				■	■									■						5	25.0
	T2					■																	
	T3			■		■																	
Pantoea spp.	T1											■	■							■		4	20.0
	T2																						
	T3																	■					
Pantoea agglomerans	T1	■					■	■	■		■	■	■									8	40.0
	T2								■														
	T3	■		■																			
Pasteurella pneumotropica	T1														■			■				4	20.0
	T2																			■			
	T3																■						
Pasteurella spp.	T1																				■	3	15.0
	T2																		■	■			
	T3																						
Salmonella spp.	T1																					1	5.0
	T2																						
	T3												■										
Serratia plymuthica	T1																					2	10.0
	T2	■									■												
	T3	■																					
Serratia marcescens	T1																					1	5.0
	T2									■													
	T3																						
Shigella spp.	T1								■													4	20.0
	T2				■					■							■						
	T3																						
Staphylococcus spp.	T1																					1	5.0
	T2	■																					
	T3																						
Streptococcus spp.	T1			■																		1	5.0
	T2																						
	T3																						
Stenotrophomonas maltophilia	T1								■	■	■			■					■	■		9	45.0
	T2												■					■	■				
	T3										■		■				■		■				

Yersinia Pestis	T1<br>T2<br>T3		1	5.0

T1 (zero hours of fermentation). T2 (middle of the fermentation time). T3 (end of the fermentation time).

Supplementary Table S7. High throughput sequencing of ITS with the MiSeq platform from Ilumina and richness and diversity indices during coffee fermentation in 20 farms department of Cesar, Colombia.

Farm	Fermentation Time (h)	Read Count	Q30 (%)	nseqs	Coverage	Genus-Species Observed (OTUs)	ACE	Shannon	Simpson
F1	0	264,51	86.40	73912	1.00	285	180.14	1.19	0.47
	18	294.06	87.50	75590	1.00	579	499.39	2.51	0.82
	36	241.83	88.50	54916	1.00	319	182.92	1.98	0.75
F2	0	215.12	87.30	54480	0.99	573	471.65	1.72	0.55
	18	316.11	89.30	93557	1.00	453	645.86	0.92	0.32
	36	230.46	88.70	57637	1.00	343	276.52	1.33	0.50
F3	0	204.37	84.00	51124	1.00	367	1427.46	0.94	0.36
	10	243.03	85.40	62868	1.00	447	312.50	1.21	0.43
	20	215.46	85.00	50101	1.00	378	1231.31	1.33	0.47
F4	0	233.13	78.50	39420	0.99	629	1844.76	2.39	0.70
	9	229.81	82.80	47070	1.00	398	1127.93	2.13	0.80
	18	209.28	84.40	47881	0.99	541	2155.69	2.30	0.79
F5	0	246.65	80.30	40141	0.99	465	1498.72	2.75	0.86
	9	243.72	81.50	44438	0.99	505	1751.26	2.71	0.87
	18	240.07	85.00	48749	0.99	424	1760.97	2.26	0.74
F6	0	239.76	83.20	53122	1.00	472	406.61	2.14	0.78
	21	282.80	87.50	76501	1.00	417	349.86	1.61	0.56
	42	265.28	89.10	70627	1.00	282	270.08	1.42	0.62
F7	0	152.35	76.90	11242	0.99	188	420.30	1.20	0.44
	18	170.88	76.00	13390	0.99	177	377.60	1.20	0.47
	36	190.06	76.60	13135	0.99	184	428.80	1.00	0.34
F8	0	207.50	78.60	36781	0.99	195	397.50	2.10	0.73
	9	218.63	80.20	47866	0.99	119	229.10	1.20	0.43
	18	216.11	81.60	47734	0.99	71	986.90	1.10	0.52
F9	0	209.08	77.90	39784	0.99	163	384.80	2.00	0.78
	9	198.04	82.10	52060	0.99	78	127.70	1.10	0.48
	18	205.34	81.20	43044	0.99	92	243.80	1.20	0.59
F10	0	181.31	79.20	33151	0.99	203	555.70	2.20	0.81
	9	224.50	81.30	51075	0.99	88	153.50	1.30	0.56
	18	210.54	81.70	48125	0.99	96	180.30	1.30	0.56
F11	0	251.14	79.40	46909	0.99	108	176.90	2.00	0.79
	18	243.23	79.80	42525	0.99	123	275.70	1.60	0.73
	36	261.52	81.00	40143	0.99	110	252.90	1.60	0.69
F12	0	190.38	78.50	40863	0.99	173	463.30	1.50	0.54

	7	207.15	81.20	52436	0.99	82	141.50	0.80	0.32
	14	228.74	81.90	59603	0.99	61	133.20	0.60	0.25
F13	0	195.78	75.00	27329	0.99	205	412.30	2.10	0.74
	8	190.70	78.70	41181	0.99	148	413.80	1.10	0.42
	16	212.15	81.60	47208	0.99	68	109.20	1.30	0.56
F14	0	244.90	76.80	38659	0.99	204	548.30	2.20	0.83
	5	263.88	80.00	48442	0.99	118	223.50	1.80	0.72
	10	252.09	81.90	55383	0.99	79	131.30	1.50	0.65
F15	0	210.91	73.10	21984	0.99	256	713.90	2.10	0.77
	8	174.63	76.10	20722	0.99	242	554.90	2.00	0.76
	16	228.12	80.10	38520	0.99	138	311.50	1.90	0.73
F16	0	244.11	75.90	40329	0.99	177	323.40	1.80	0.70
	7	281.36	75.70	37816	0.99	161	305.40	2.30	0.83
	14	213.52	79.00	35953	0.99	136	223.80	2.10	0.67
F17	0	242.82	79.50	39726	0.99	150	383.40	2.00	0.80
	8	200.71	79.50	34202	0.99	138	324.30	2.10	0.79
	16	231.17	79.60	39525	0.99	132	382.70	1.70	0.72
F18	0	212.76	81.00	47012	0.99	77	127.30	1.50	0.60
	9	202.67	80.80	50092	0.99	81	192.60	1.10	0.44
	18	217.59	81.00	49098	0.99	68	105.30	1.20	0.47
F19	0	224.89	77.80	39283	0.99	213	491.50	2.40	0.81
	9	245.74	80.50	54517	0.99	112	220.00	1.40	0.53
	18	267.52	80.70	52501	0.99	79	136.80	1.30	0.57
F20	0	225.75	79.00	38768	0.99	183	387.70	2.30	0.82
	9	282.29	80.10	55371	0.99	129	294.00	2.00	0.78
	18	253.12	81.20	50023	0.99	84	184.50	1.70	0.71

Supplementary Table 8S. Identification and frequency of yeasts isolated into pure culture in coffee mucilage during the fermentation process at three different times in the 20 studied farms.

Microorganism	Time	Farms																				Frequency by farms	
		F1	F2	F3	F4	F5	F6	F7	F8	F9	F10	F11	F12	F13	F14	F15	F16	F17	F18	F19	F20	#	%
Candida tropicalis	T1	■											■	■		■		■	■			9	45.0
	T2	■						■				■	■	■		■	■	■	■				
	T3												■	■		■	■		■				
Candida rugosa	T1	■							■	■	■								■	■		6	30.0
	T2																		■	■			
	T3																			■			
Candida guillermondii	T1	■	■				■	■	■	■		■		■							■	10	50.0
	T2	■						■	■	■	■	■		■			■						
	T3	■	■				■	■	■	■	■	■		■									
Cryptococcus laurentii	T1	■																				4	20.0
	T2	■									■												
	T3		■									■											
Crytococcus neoformas	T1							■	■							■						6	30.0
	T2	■						■	■	■		■											
	T3	■						■	■							■							
Hanseniaspora	T1			■	■			■	■	■	■	■		■	■	■	■	■	■	■		15	75.0
	T2			■	■	■		■	■	■	■	■		■	■	■	■	■	■	■			

	T3		
Candida albicans	T1		
	T2	11	55.0
	T3		
Candida kefyr	T1		
	T2	8	40.0
	T3		
Candida dubliniensis	T1		
	T2	1	5.0
	T3		
Candida parapsilosis	T1		
	T2	3	15.0
	T3		
Candida lipolytica	T1		
	T2	2	10.0
	T3		
Candida kruesi	T1		
	T2	10	50.0
	T3		
Rhodotorula glutinitis	T1		
	T2	2	10.0
	T3		
Candida zeylanoides	T1		
	T2	1	5.0
	T3		
Rhodoturalla spp	T1		
	T2	3	15.0
	T3		
Rhodoturalla mucilaginosa	T1		
	T2	3	15.0
	T3		
Saccharomyces cerevisiae	T1		
	T2	1	5.0
	T3		
Pichia guillermondii	T1		
	T2	1	5.0
	T3		
Pichia sp	T1		
	T2	2	10.0
	T3		
Candida glabrata	T1		
	T2	1	5.0
	T3		

T1 (zero hours of fermentation). T2 (middle of the fermentation time). T3 (end of the fermentation time).

Supplementary Table S9. Identification and frequency of mycelial fungi isolated into pure culture in coffee mucilage during the fermentation process at three different times in the 20 studied farms.

Microorganism	Time	Farms																				Frequency by farms	
		F1	F2	F3	F4	F5	F6	F7	F8	F9	F10	F11	F12	F13	F14	F15	F16	F17	F18	F19	F20	#	%
Alternaria sp	T1																						
	T2																				■	1	5.0
	T3																						
Aspergillus aureolus	T1																						
	T2																					1	5.0
	T3															■							
Aspergillus flavus	T1	■					■								■						■		
	T2	■											■		■						■	5	25.0
	T3														■								
Aspergillus fumigatus	T1																■	■					
	T2											■					■	■			■	5	25.0
	T3															■	■	■					
Aspergillus glaucus	T1	■																					
	T2																					1	5.0
	T3																						
Aspergillus niger	T1			■												■							
	T2			■																		2	10.0
	T3			■																			
Aspergillus oryzae	T1	■																					
	T2																					1	5.0
	T3																						
Aspergillus terreus	T1											■			■								
	T2											■			■							3	15.0
	T3				■										■								
Beauveria spp	T1																						
	T2																					1	5.0
	T3			■																			
Cladosporium spp	T1																■						
	T2		■														■			■	■	4	20.0
	T3																■				■		
Curvularia spp	T1																						
	T2			■																		1	5.0
	T3																						
Fusarium spp	T1												■										
	T2											■										2	10.0
	T3																						
Geotrichum spp	T1									■													
	T2	■								■												2	10.0
	T3									■													
Mucor spp	T1	■									■												
	T2																				■	3	15.0
	T3																				■		
Neocytalidium spp	T1											■											
	T2															■						3	15.0
	T3															■					■		
Penicillium chrysogenum	T1																						
	T2																					1	5.0
	T3	■																					
Penicillium spp	T1			■			■					■				■		■		■	■		
	T2			■			■				■					■	■	■				9	45.0
	T3						■											■					
Phytophthora spp	T1		■																				
	T2																					1	5.0
	T3																						

Rhizopus spp	T1	■		■	■								■	■			5	25.0
	T2	■		■	■								■	■				
	T3	■												■				
Scopulariopsis spp	T1										■						1	5.0
	T2																	
	T3																	
Sporotrichum spp	T1																1	5.0
	T2															■		
	T3																	
Syncephalastrum spp	T1													■			1	5.0
	T2													■				
	T3													■				
Trichoderma spp	T1					■			■								2	10.0
	T2					■			■									
	T3					■												

T1 (zero hours of fermentation). T2 (middle of the fermentation time). T3 (end of the fermentation time).

Supplementary Table S10. Total SCA score and sensory descriptors in farms from the department of Cesar.

Farm	Fermentation Time (h)	Humidity (%)	Clean cup	Total SCA Score	Sensory Description
F1	0	12.0	10.0	81.74	Herbal, chocolat, brown sugar
	18	12.3	0.0	53.00	Ferment
	36	9.9	0.0	53.63	Mold
F2	0	8.6	10.0	82.38	Brown sugar, nuts, cereal
	18	8.7	10.0	83.13	Chocolat, brown sugar, citrus floral, herbal
	36	8.8	10.0	84.25	Apple, fruity, honey.
F3	0	9.1	10.0	82.74	Nuts, cereal, citrus, vanilla. Fine acidity light body
	10	9.0	10.0	84.00	Positive herbal, black Tea, blackberries, brown sugar. Fine acidity. Juicy body
	20	10.2	10.0	81.78	Cinnamon, caramel. Medium acidity. Low body. Dry residual.
F4	0	11.8	10.0	80.50	Brown sugar, cereal. Dry residual. Light body
	9	11.3	10.0	83.00	Cereal, pea, citrus, honey, caramel. Skinny body. Medium fine acidity
	18	12.1	10.0	83.25	Fruity, herbal, citrus, brown sugar, honey. Delicate acidity. Juicy body. Balanced
F5	0	9.1	10.0	83.00	Brown sugar, caramel, citrus, chocolate
	9	10.1	10.0	82.74	Nuts, cereal, citrus, vanilla. Fine acidity. Light body
	18	10.1	10.0	82.49	Brown sugar, caramel.
F6	0	9.5	10.0	85.27	Floral, fruity, red fruits, cherry. Bright acidity. Smooth body
	21	10.5	10.0	84.64	Fruity flavor, red fruits, apple. Bright acidity. Creamy body
	42	9.5	10.0	83.74	Caramel, blackberries, cherry. Clean residual. Low acidity. Light body
F7	0	11.2	10.0	81.50	Herbal, brown sugar, citrus
	18	11.3	10.0	81.50	Citrus, red fruits, honey, acidity, astringent, flat cup

	36	11.1	10.0	84.00	Red fruits, blackberries, fruity, caramel, citrus, brown sugar. Dry residual. Balanced and clean body
F8	0	9.8	10.0	81.75	Sweet, straw, citrus
	9	10.6	10.0	83.13	Citrus, floral, jasmine, chocolat, caramel, walnut. Acidity and medium body
	18	10.4	10.0	83.63	Caramel, citrus, brown sugar, honey. Balanced body
F9	0	11.3	10.0	80.88	Herbal, nuts
	9	11.4	10.0	81.50	Brown sugar, chocolat, cereal, flat cup
	18	11.5	10.0	81.50	Herbal, brown sugar, dry. Low acidity, cereal
F10	0	11.2	10.0	82.50	Chocolate, brown sugar, caramel, citrus
	9	11.5	10.0	82.25	Brown sugar, chocolat
	18	11.3	10.0	82.13	Citrus, caramel, brown sugar, honey
F11	0	11.4	10.0	81.50	Herbal, brown sugar, citrus
	18	11.2	10.0	81.50	Citrus, brown sugar, caramel
	36	10.7	10.0	83.00	Vanilla, citrus, herbal, cane. Fine acidity. Residual straw
F12	0	10.3	10.0	81.50	Herbal, honey
	7	10.4	10.0	82.75	Caramel, citrus, brown sugar, species. Residual cereal
	14	11.4	10.0	81.63	Herbal, cereal. Low acidity. Medium and light body
F13	0	9.8	10.0	83.00	Caramel, brown sugar, citric, fruity, blackberries
	8	10.2	10.0	82.75	Brown sugar, red fruits, citrus
	16	11.0	10.0	83.63	Brown sugar, citric, chocolate, spicy
F14	0	9.8	10.0	81.88	Citrus, herbal, caramel, cereal
	5	10.2	10.0	82.00	Herbal, brown sugar, citrus
	10	10.0	10.0	81.75	Herbal, citrus, brown sugar. Dry and rough residual
F15	0	9.0	10.0	82.75	Citrus, caramel, fruit, brown sugar
	8	10.6	10.0	83.25	Caramel, floral, citrus, floral
	16	10.6	10.0	83.50	Citrus, floral, caramel
F16	0	10.6	10.0	83.50	Caramel, chocolat, brown sugar, honey, floral, rose, tea. Consistent balance
	7	10.2	10.0	82.50	Citrus, brown sugar, herbal, nuts, honey. Low acidity.
	14	10.1	10.0	82.50	Nuts, herbal, cereal fragrance, citrus, medium herbal background. Light body
F17	0	10.7	10.0	83.63	Brown sugar, citrus, honey

	8	9.7	10.0	83.00	Chocolat, caramel, brown sugar, nuts. Clean residual. Medium acidity
	16	10.1	10.0	82.13	Yellow fruits, citrus, caramel, fruit flavor. Dry residual
F18	0	11.2	10.0	81.13	Caramel, chocolat, nuts
	9	11.0	10.0	82.38	Nuts, herbal, brown sugar, citric. Acidity and medium body
	18	10.7	10.0	82.13	Citrus, caramel, herbal, flat cup, cereal. Dry residual
F19	0	11.6	10.0	85.13	Caramel, citrus, spicy, balanced, clean residual, fruity, grapes, cane. Juicy body
	9	11.6	10.0	83.13	Citrus, orange, brown sugar, yellow fruits, residual straw-cereal. Fine acidity. Juicy body
	18	11.8	10.0	83.13	Citrus, caramel, brown sugar. Medium acidity. Dry and cereals residual.
F20	0	11.2	10.0	83.13	Citrus, brown sugar, caramel
	9	12.1	0.00	52.00	Contaminated
	18	10.7	10.0	82.75	Red fruits, citrus, orange, chocolate. Dry residual. Acidity and body medium.

*Total SCA score: 90-100 Outstanding, 85.0-89.99 Excellent, 80-84.99 Very Good, <80 Commercial Coffee.
